# Assessment of Significant Factors Affecting Frequent Lane-Changing Related to Road Safety: An Integrated Approach of the AHP–BWM Model

**DOI:** 10.3390/ijerph182010628

**Published:** 2021-10-11

**Authors:** Danish Farooq, Sarbast Moslem, Arshad Jamal, Farhan Muhammad Butt, Yahya Almarhabi, Rana Faisal Tufail, Meshal Almoshaogeh

**Affiliations:** 1 Department of Civil Engineering, Comsats University Islamabad, Wah Campus, Wah 47040, Pakistan; danish.farooq@ciitwah.edu.pk (D.F.); faisal.tufail@ciitwah.edu.pk (R.F.T.); 2 Department of Transport Technology and Economics, Budapest University of Technology and Economics, 1111 Budapest, Hungary; moslem.sarbast@mail.bme.hu; 3 Department of Civil and Environmental Engineering, College of Design and Built Environment, King Fahd University of Petroleum & Minerals, KFUPM Box 5055, Dhahran 31261, Saudi Arabia; arshad.jamal@kfupm.edu.sa; 4 Interdisciplinary Research Center of Smart Mobility and Logistics (IRC-SML), King Fahd University of Petroleum & Minerals, Dhahran 31261, Saudi Arabia; 5 Transportation and Traffic Engineering Department, College of Engineering, Imam Abdulrahman Bin Faisal University, P.O. Box 1982, Dammam 31451, Saudi Arabia; 6 Center of Excellence in Trauma and Accidents, King Abdulaziz University, Jeddah 21589, Saudi Arabia; Yalmarhabi@kau.edu.sa; 7 Department of Surgery, Faculty of Medicine, King Abdulaziz University, Jeddah 21589, Saudi Arabia; 8 Department of Civil Engineering, College of Engineering, Qassim University, Buraydah 51452, Saudi Arabia; m.moshaogeh@qu.edu.sa

**Keywords:** frequent lane-changing, road safety, prioritize, multi-criteria decision making, analytic hierarchy process, best–worst method

## Abstract

Frequent lane changes cause serious traffic safety concerns for road users. The detection and categorization of significant factors affecting frequent lane changing could help to reduce frequent lane-changing risk. The main objective of this research study is to assess and prioritize the significant factors and sub-factors affecting frequent lane changing designed in a three-level hierarchical structure. As a multi-criteria decision-making methodology (MCDM), this study utilizes the analytic hierarchy process (AHP) combined with the best–worst method (BWM) to compare and quantify the specified factors. To illustrate the applicability of the proposed model, a real-life decision-making problem is considered, prioritizing the most significant factors affecting lane changing based on the driver’s responses on a designated questionnaire survey. The proposed model observed fewer pairwise comparisons (PCs) with more consistent and reliable results than the conventional AHP. For level 1 of the three-level hierarchical structure, the AHP–BWM model results show “traffic characteristics” (0.5148) as the most significant factor affecting frequent lane changing, followed by “human” (0.2134), as second-ranked factor. For level 2, “traffic volume” (0.1771) was observed as the most significant factor, followed by “speed” (0.1521). For level 3, the model results show “average speed” (0.0783) as first-rank factor, followed by the factor “rural” (0.0764), as compared to other specified factors. The proposed integrated approach could help decision-makers to focus on highlighted significant factors affecting frequent lane-changing to improve road safety.

## 1. Introduction

The Global status report on road safety estimated that the number of fatalities in annual road traffic accidents have reached over 1.35 million [1]. European roads have been declared as the safest worldwide, with a decline of 19% in road fatalities over the previous six years. While attaining the strategic goal of halving the number of road crashes fatalities between 2010 and 2020 is an acute task, it is worth aiming to protect every single life [2]. In addition, the performance of Hungary’s road safety is below the EU average. On Hungarian roads, 64 people per million inhabitants died in 2018, indicating a 1% rise compared to the previous year [3]. The situation analysis of the Road Safety Action Program examines that human-related issues cause most of the road accidents; thus, controlling them becomes the extremely dynamic goal of road safety actions [4,5,6]. Previous findings detected the human factors to be a primary or leading contributing cause in approximately 90% of road traffic crashes [7,8,9,10,11,12]. In addition, the study observed that driving behavior is one of the basic driver-related components that directly affect road safety [13,14,15].

Frequent lane-changing actions have a negative impact on traffic performance, particularly, when performed during high traffic exposure periods [16]. A study report [17] stated that the action of changing lanes is one of the highly frequent sources of crashes in the United States. Accordingly, the official statistics estimated that at least 33% of all road crashes happen as vehicles alter lanes or turn off the road. Furthermore, crash data recorded from 2010 to 2017 in Middle East countries indicate that sudden lane changes produced about 17.0% of the total serious accidents, followed by speeding (12.8%) [18,19].

In recent years, many researchers studied the lane-changing phenomenon using different statistical and dynamic techniques. Accordingly, some researchers implemented game theory to examine lane-changing behavior [20,21,22,23]. In addition, the good reputation of artificial intelligence (AI) has preceded some AI-based lane-changing behavior investigations. The neural network model can estimate lane-changing behavior more precisely than the multinomial logit model [24]. Probabilistic methods were utilized to analyze lane-changing behavior [25]. Researchers further developed an exponential probability model to examine the relationship between lane-changing probability and relative gap, as well as relative velocity. Furthermore, a recent study utilized a simulation model to evaluate the influence of significant factors on lane-changing [26].

Studying the unpredictability of the lane-changing period, a previous study [27] investigates that the least possible safety layout alters with the dynamic lane-changing period and explains how the accelerating space of the actual vehicle alters with the nearby vehicles’ running positions. On this basis, a decision-making model of discretionary lane-changing based on dynamic reference spots was created using cumulative prospect theory (CPT). This CPT-based model can depict discretionary lane-changing behavior in a more precise way, which reflect drivers’ risk abhorrence during decision-making. Furthermore, another study [28] suggested a new technique to formalize the lane-changing model in urban driving situations. They describe human incentives from different angles, route-change incentives, speed incentive, courtesy incentive and comfort incentive, etc. Furthermore, a decisional theoretical tool was employed, called multi-criteria decision-making (MCDM), to consider these incentive strategies. The strategy of grouping is in accordance with different driving styles, which differ for each driver. Therefore, a lane-changing decision selection algorithm was suggested. The proposed model could identify more abundant and unexpected lane-changing behaviors with different preferences of inducements.

A previous study depicted that many experts applied the analytic hierarchy process (AHP) to compare the decisions by utilizing their proficiencies and intellectual resources; however, this may not entirely replicate the approach of human behavior. In addition, the inconsistency in pairwise comparison matrices (PCMs) is the basic shortcoming of AHP and can yield biased results. However, it is generally evident that, if the PCM is 5 × 5 or greater in the decision arrangement, the comparatively reliable filling of a matrix of this size by non-expert assessors needs substantial endeavor [29,30,31]. After the development of the best–worst method (BWM), the investigators have commenced to utilize that instead of the AHP because of its advantages. The BWM/F-BWM is employed to allocate weights to risk factors similar to the AHP [32]. In several studies, it is often integrated with the failure mode and effects analysis (FMEA) [33,34,35,36]. For the BWM, the inconsistency-improving actions and the suitable consistency ratio value can be tackled through input- and output-based consistency techniques. In addition, the BWM, within other situations, could examine the ambiguity. In addition, in the BWM, the model’s multiple optimality result can be estimated from other perspectives [37,38]. Recently, a study provided a perfect and clear approach for evaluating and ranking suppliers based on a consistent, consensus-derived model for multi-criteria group decision-making (MCGDM) in a partial AHP condition [39]. Sometimes experts may not be capable of selecting the most appropriate criterion weight vector in the logic of intuitionist 2-tuple linguistic (I2TL) sets for multicriteria decision-making (MCDM). To elude this condition, the decision-makers (DMs) can utilize the best–worst method (BWM), in which DMs select the best (most important) criterion and the worst (least important) criterion and then give two preference vectors by relating the best criterion to one vector and the worst criterion to the other [40]. Pythagorean fuzzy sets (PFSs) were observed to be a superior technique introduced for multi-criteria decision-making, facilitating both membership and non-membership functions in a big domain region [41]. Furthermore, another study [28] suggested a new technique to formalize the lane-changing model in urban driving situations. They describe human incentives from different angles, route-change incentive, speed incentive, courtesy incentive and comfort incentive, etc. A decisional theoretical tool was employed, called multi-criteria decision making (MCDM), to consider these incentive strategies. Mainly, the strategy of grouping was undertaken according to different driving styles, which differ for each driver. Therefore, a lane-changing decision selection algorithm was suggested. The proposed model identified more abundant and unexpected lane-changing behaviors with different preferences of inducements [28].

In recent crash reports, it is generally recorded that the crash has happened because of the frequent-lane-changing behavior of the at-fault drivers (in the crash reason item), without considering the factors underlying such behavior [42,43]. Most of the previous findings concentrated on significant driver behavior factors that could affect road safety [13,44,45], but these papers lack specific estimation of the most significant factors and sub-factors that could affect frequent lane-changing. Therefore, this study considers those factors that could affect the occurrence of frequent lane-changing while investigating the drivers’ perception towards lane change maneuvering.

The main goal of the present study is to identify and rank the significant factors affecting lane-changing by utilizing an integrated model of AHP and BWM. For this research study, we designed a lane-change model consisting of important factors and their sub-factors in a three-level hierarchical arrangement for evaluation purposes. In addition, the proposed model requires a smaller number of PCs with more reliable outcomes than the classical AHP in real-life decision-making problems. The research work is structured as follows: The exhaustive steps of the questionnaire survey with the focus on the AHP and BWM design are presented in Section 1. Afterward, the real-life case study on the application of the proposed model is presented; we evaluate the main factors and sub-factors affecting frequent lane-changing in the designed three-level hierarchical arrangement related to road safety. Then, in the next section, we present a detailed description of the AHP–BWM model and the methodological principles and characteristics of our created AHP–BWM model. In the results section, the study prioritized the specified lane-change factors by assigning weights to each factor. Finally, we draw some conclusions and make some recommendations to the users of the proposed methodology, along with remarks for further research.

## 2. Materials and Methods

### 2.1. Questionnaire Survey

There has been considerable work undertaken to identify and solve the actions that reduce driving safety. The Driver Behavior Questionnaire (DBQ) stands out for its longevity and leading use among the several tools [46]. To estimate problematic driving behavior, the Driver Behavior Questionnaire (DBQ) was first utilized as a means in the relevant studies in the 1990s [47]. The current study applied the questionnaire survey designed on a Saaty scale to evaluate the effect of significant factors on frequent lane-changing based on drivers’ responses. Accordingly, the questionnaire was designed into two parts. The first part consists of important socio-demographic information related to participants, such as age, gender, duration of driving license, education level and working status, as shown in Table 1. These characteristics affect the driver’s perception towards lane-changing maneuvers [42,48]. The second part of questionnaire survey consists of questions about significant factors affecting frequent lane-changing designed for the AHP model and the BWM model, as shown in Table 2 and Table 3. To collect questionnaire data, the survey was distributed among seventy participants from the department of transport technology and economics (Budapest University of Technology and Economics) who had a driving license; the respondents were asked to fill the survey through Google online forms, etc. The response rate was 70%. The evaluator number of the questionnaire survey was not statistically representative, while Solomon [49] highlighted, in his phenomenon “Wisdom of crowds,” that 20 evaluators can provide an extreme opinion.

### 2.2. Lane-Change Model

**Figure 1:**
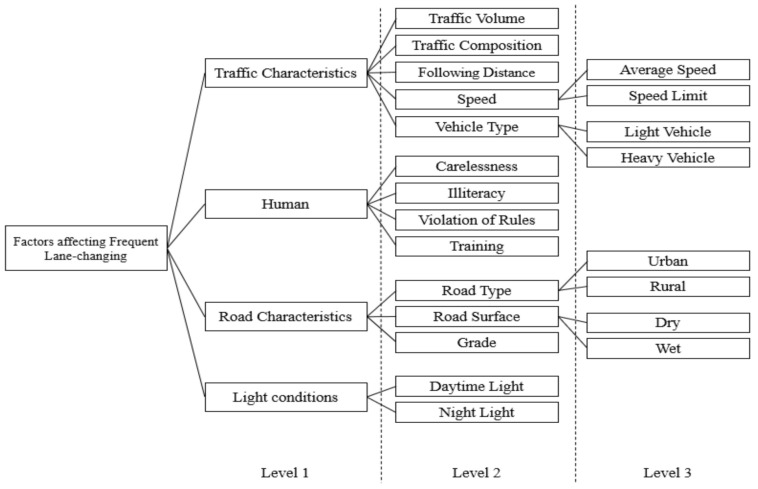
Three-level hierarchical structure of the factors affecting frequent lane-changing (Lane-change model).

The lane-changing trend is defined as “an act of driving practice that changes a vehicle from one lane to another as both lanes get the same way of travel” [50]. In this research study, we designed a lane-change model consisting of important factors and their sub-factors in a three-level hierarchical arrangement for evaluation purposes, as presented in Figure 1. The first level consists of four primary factors related to road safety, such as traffic characteristics, human, road characteristics and light conditions. A descriptive–analytic study observed three similar main categories of factors that contribute to crashes caused by fatigue and sleepiness, such as human, road and light conditions and vehicle-related factors [51]. For level 2, these main factors were further distributed into fourteen sub-factors and, for level 3, these were further distributed into eight sub-factors. Many researchers considered these specified factors significant in related studies, such as lane-change crashes [42,52], lane-change risks [53], lane-changing simulation [26], discretionary lane-changing [27,54] and road-safety evaluation [44].

### 2.3. Analytic Hierarchy Process (AHP)

AHP is one of the most widely applied MCDM methodologies in engineering, technology, science, and management and business [55,56,57,58]. AHP helps decision-makers estimating complex problems with several differing and biased measures. In the AHP domain, several combined strategies are proposed [42]. The evaluator in the survey sometimes ought not to give a numerical judgment; as an alternative, a relative verbal comprehension, more typical in our everyday lives, is enough [57]. However, the 1–9 fundamental scale proposed by Saaty et al. [59] is commonly applied to evaluate the factors by considering PCs. The main steps of the AHP are:

Step 1: Determining the hierarchical structure of the evaluation factors.

Step 2: Constructing the survey based on PCs in the hierarchical structure by using a (1–9) scale.

Step 3: Checking the consistency of PCs.

Step 4: Aggregating the weight scores.

Step 5: Deriving weight vectors and computing the final weight scores considering branch connections.

Step 6: Performing the sensitivity analysis.

In the AHP domain, several combination strategies are proposed. The evaluator in the survey sometimes does not require to give a numerical judgment; instead of relative verbal gratitude, more typical in our daily life, is enough. However, a 1–9 fundamental scale was proposed by Saaty (1977) to evaluate the PCs numerically or verbally (Table 4). The conducted comparisons are estimated in a positive reciprocal matrix (1).

(1) $$X=\left[\begin{array}{cc} \begin{array}{cc} x_{ij} & x_{12}\\ x_{21} & 1 \end{array} & \begin{array}{cc} \ldots & x_{1n}\\ \ldots & : \end{array}\\ \begin{array}{cc} : & \ldots \\ x_{n1} & \ldots \end{array} & \begin{array}{cc} 1 & :\\ \ldots & 1 \end{array} \end{array}\right]\;$$

where $x_{ij}$ is the comparison between factor *i* and factor *j* and *n* is the dimension of the PCM and, at the same time, it is the number of evaluated factors in the matrix; the total number of comparisons is $n\left(n-1\right)/2$.

If the matrix is perfectly consistent, then $x_{ij}=x_{in}.x_{nj}$, for checking matrix consistency. The matrix consistency is entirely consistent and acceptable when *CR* is smaller than 10%, else the evaluator has to re-evaluate the PCM. Saaty (1977) adopted the consistency index (*CI*), which is related to the maximum eigenvalue $\lambda_{max}$:

(2) $$CI=\frac{\lambda_{max}-n}{n-1}$$

The consistency ratio is computed by

(3) CR=CI/RI

where RI is the random index, as given in Table 5.

### 2.4. Best–Worst Method (BWM)

The developed BWM considers an efficient MCDM method to deal with complex problems. In this approach, the decision-maker picks the most important criterion and the least important one from all considered criteria. Then, the criterion selected as the best or the most important is compared with all the other criteria and allocated a number in the range between 1 and 9, where “1” characterizes that the criterion is as significant as the best or most significant criterion and “9” characterizes that the finest criterion is nine times more significant than the worst criterion [29]. Similarly, the comparison is performed among the criterion selected as the worst or the least important and all the other criteria. These values are then managed for an optimization process [38]. The main aim of these comparisons is to obtain the maximum consistency by reducing the maximum difference between the best-criterion-to-any-criterion comparison value and the proportion of the weight scores of the best criterion and that criterion. This process is performed by certain new value constraints, such as the maximum potential value of the sum of the weight scores. For evaluating the factors’ weight scores, a 2n−3 comparison number is required. The efficiency of the method made the scholars adopt it in several studies in a short period [32,60,61]. The main steps of the method are as follows:

Step 1: Setting up the problem structure of factors.

Step 2: Selecting the most critical factor and the least critical factor based on the evaluator’s point of view.

Step 3: Comparing the most critical factor with other factors employing a 1–9 scale; the result would be the most important to other factors: $X_{b}=\left(x_{b1},\;x_{b2},\;\ldots ,x_{bn}\right),\;$ where $x_{bj}$ is the preference of the most critical factor *b* over factor *j* and $x_{bb}=1$.

Step 4: Comparing the least important factor with other factors employing a 1–9 scale; the result would be the least important factor to other factors: $X_{w}={\left(x_{1w},\;x_{2w},\;\ldots ,x_{nw}\right)}^{T},\;$ where $x_{jw}$ is the preference of factor *j* over the least important factor *w* and $x_{ww}=1$.

Step 5: Calculating the optimal weight scores ($W_{1}^{*},\;W_{2}^{*},\;\ldots ,\;W_{n}^{*}$) by minimizing the maximum between the $\left|W_{b}-x_{bj}.W_{j}\right|\;$ and $\left|W_{j}-x_{jw}.W_{w}\right|$ as follows:

(4) $$min.max.\;\left\{\left|W_{b}-x_{bj}.W_{j}\right|,\left.\left|W_{j}-x_{jw}.W_{w}\right|\right\}\right.,\;s.t.\;\sum_{j=1}^{n}W_{j}=1\;W_{j}\;\geq 0,\;\forall \;j$$

To measure the PC consistency in the BWM, the input-based consistency ratio ($CR^{I}$) by [62] was adopted. The new measurement technique can provide the results immediately by using the evaluator’ preferences without calculating the entire optimization and it could be calculated as follows:

(5) $$CR^{I}=\underset{j}{\max}CR_{j}^{I}\;$$

where

(6) $$CR_{j}^{I}=\left\{\begin{array}{c} \frac{\left|x_{bj}\times x_{jw}-x_{bw}\right|}{x_{bw}\times x_{bw}-x_{bw}}\\ 0 \end{array}\right.\;\;\;\;\;\;\;\;\begin{array}{c} x_{bw}>1\\ x_{bw}=1 \end{array}.$$

The proposed measurement illustrates how much an evaluator disregards the ordinal consistency and it gives an appropriate process to highlight and fix the included conflicts. The threshold of the input-based consistency is obtained from Table 6.

Step 7: Aggregating the weight scores.

Step 8: Deriving weight vectors.

Step 9: Performing the sensitivity analysis.

### 2.5. The Proposed AHP–BWM Model

The main intention of the proposed model’s application is to obtain more reliable outcomes than those derived by the traditional model in multi-level complex decision problems. The model efficiency comes from providing consistent PCs and minimizing the comparison number in the conducted surveys. In the AHP approach, the consistency would be very weak if the factor number in the PCM leaped 7 ± 2 Saaty (1977). However, Rezaei [38] created the BWM approach to secure this gap and provide comparisons that are more consistently efficient. Moreover, the BMW requires a smaller number of comparisons (2*n* − 3) than the AHP (*n*(*n* − 1)/2) when factor numbers are 4 or greater.

These matters show that the BWM approach is an effective method to conserve time and energy for both assessors and decision-makers. For example, if we have only 20 factors, that means the evaluator needs to make just 37 comparisons with the BWM. On the other hand, the evaluator needs to make 190 comparisons with the conventional AHP approach.

The main steps of the AHP–BWM model are as follows:

Step 1: Creating the hierarchical structure of the complex problem.

Step 2: Determining factor numbers for each cluster in the hierarchical structure.

Step 3: Adopting the AHP method for all clusters with four factors and smaller to compute the factors’ weight scores and checking the consistency.

Step 4: Adopting the BWM method for all clusters with five factors and bigger to compute the factors’ weight scores and checking the consistency.

Step 5: Aggregating the weight scores.

Step 6: Conducting the final weight scores considering branch connections.

Step 7: Performing the sensitivity analysis.

## 3. Results and Discussion

The AHP–BWM model was adopted based on the number of factors in each cluster to estimate factors significance affecting frequent lane-changing based on drivers’ responses. The total number of evaluated comparisons for the conventional AHP was 20 (12 comparisons for 2 (4 × 4) matrices + 5 comparisons for 5 (2 × 2) matrices + 3 comparisons for one (3 × 3) matrix), and 7 comparisons were evaluated for the BWM. Following that, the total number of the evaluated comparisons for the proposed model was 27 comparisons. However, if the conventional AHP was applied for all structures, the evaluator needed to evaluate 30 comparisons. The results were achieved based on the following steps:

Step 1: The hierarchical structure of the factor influencing frequent lane-changing was constructed based on the literature and driving experts’ point of view (Figure 1). The hierarchical structure of the problem consists of four main factors located in level 1, 14 sub-factors located in the second level and gathered into four groups, and eight sub-factors located in the third level and gathered into four groups.

Step 2: Determining factor numbers for each cluster in the hierarchical structure. The first level consists of one cluster with four factors. The second level consists of four clusters (1. Cluster contains five factors; 2. Cluster contains four factors; 3. Cluster contains three factors and 4. The cluster contains two factors) and the third level consists of four clusters, each containing two factors.

Step 3: The AHP method is adopted for all clusters with four factors and smaller to compute the factors’ weight scores; CR was smaller than 0.1 for all PCs.

**Figure 2:**
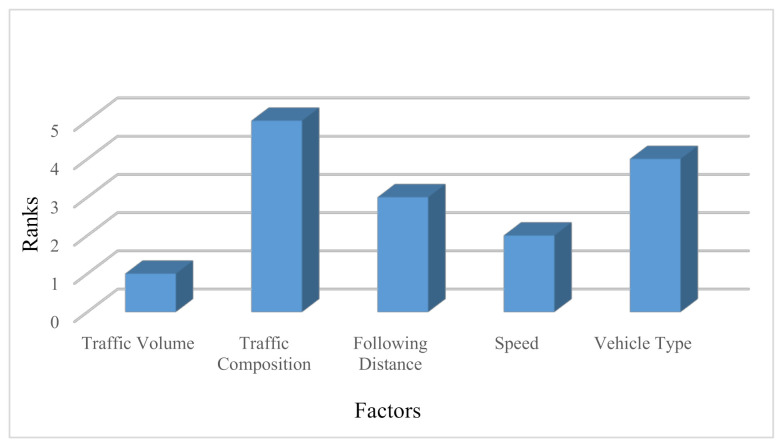
The ranks of specified factors based on final aggregated weights obtained using BWM.

Step 4: The BWM method was conducted for only one cluster with five factors (traffic volume, traffic composition, following distance, speed and vehicle type), which is located in the second level and the $CR^{I}$ was accepted for all PCs. The final aggregated weight vector was (0.3440, 0.1075, 0.1381, 0.2954, 0.1150) and the consistency of all comparisons was smaller than the input-based threshold (0.3337). The ranks of specified factors based on final aggregated weights obtained using the BWM are shown in Figure 2.

Step 5: The aggregated weight scores for all factors obtained using the geometric mean are depicted in Table 7.

**Figure 3:**
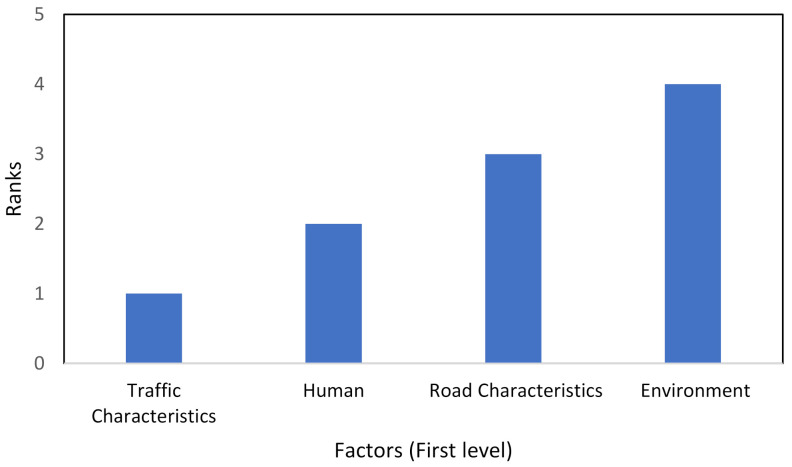
The ranks of specified factors in the first level based on the AHP approach.

**Figure 4:**
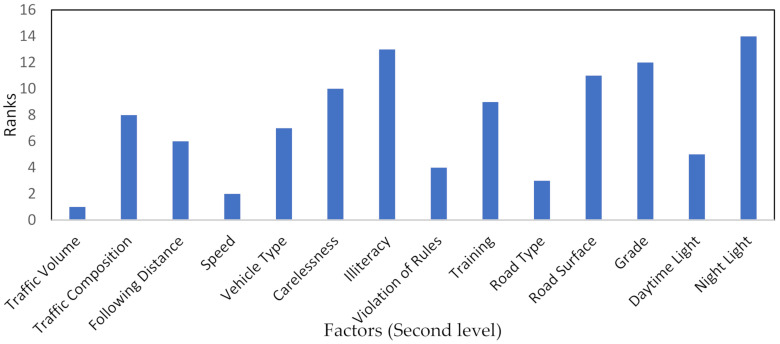
The ranks of specified factors in the second level based on the AHP–BWM model.

**Figure 5:**
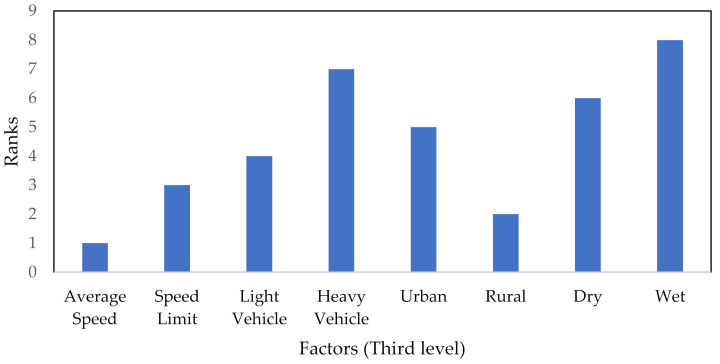
The ranks of specified factors in the third level based on the AHP–BWM model.

Step 6: The final weight scores considering branch connections are depicted in Figure 3, Figure 4 and Figure 5.

Step 7: The sensitivity analysis was also conducted.

The AHP–BWM procedure prioritized the specified factors affecting frequent lane-changing based on measured weight scores, as presented in Table 7. It is important to notice that the weights achieved using this model are comparable and the differences between the specific values are slight. For level 1, the highest weight score observed is “0.5148” for the factor “traffic characteristics” and the lowest weight score observed is “0.0884” for the factor “light conditions”. For level 2, the highest weight score observed is “0.8396” for the factor “daytime light” and the lowest weight score observed is “0.1075” for the factor “traffic composition”. For level 3, the highest weight score observed is “0.7199” for the factor “dry” and the lowest weight score observed is “0.2801” for the factor “wet”.

For level 1, the AHP procedure was applied to prioritize the specified factors affecting frequent lane-changing based on measured weight scores, as shown in Figure 3. The ranking results found “traffic characteristics” as the most significant factor, followed by “human” as a second-rank factor. A previous study observed the significant influence of stated traffic characteristics on lane-changing [63]. In comparison, the ranking results found “light conditions” as the least important factor based on evaluators’ response data. A recent study connected elevated traffic competence and safety with the least light conditional effect [64].

For level 2, the model was implemented to prioritize the observed factors affecting frequent lane-changing based on final weight scores. The model results evaluated “traffic volume” as first-rank factor, followed by “speed” as second-rank factor, as compared to other specified factors. Previous studies analyzed that traffic volume has a significant impact on the following gap and overtaking frequency [65,66], which are inherently related to lane-changing. Recent simulation-based studies found that speed has a major effect on lane-changing related to road safety, such as average speed variation, speed distribution [26], and driving speed exceeding speed limits [67]. In addition, the model results indicate “night light” as the least important factor, followed by “illiteracy” as the second least important factor. The previous study depicted that drivers are less likely to get involved in risky attitudes on multi-lane roads under bad-light situations [68]. Other factors have their ranking based on weight scores, as shown in Figure 4.

For level 3, the AHP–BWM model was also applied for prioritization of specified factors based on measured weight scores, as presented in Figure 5. The model results showed “average speed” as first-rank factor affecting frequent lane-changing. Average speed was extracted as one of the key variables of lane-change maneuvers based on the driving simulation data [69]. The factor “rural” was observed as second-rank factor, as compared to other nominated factors. In addition, a study based on crash data revealed that the proportion of lane-change crashes is higher on rural roads [11]. Furthermore, the model results indicate “wet” as the least important factor affecting frequent lane-changing, followed by “heavy vehicle” as second least important factor. A previous study found that, in light wet surface conditions, the traffic flows are smaller and the distance between vehicles (gap) is larger [70].

The study further compared the ranks of factors for the adopted AHP–BWM model and conventional AHP approach based on final measured weights. For level 2, the comparison results show the same ranks based on final weight scores for all observed factors based on specified applications, as shown in Table 8. For level 3, the comparison results for the adopted AHP–BWM model and conventional AHP approach show different ranks based on final measured weights for factors such as “average speed” and “rural” based on measured weight scores, as presented in Table 9.

In order to measure the similarity of two ratings in the MCDM methodologies, the following formula was adopted [71]:

(7) $$WS=1-\overset{n}{\underset{i=1}{\sum }}\left(2^{R_{xi}}.\;\frac{\left|R_{xi}-R_{yi}\right|}{max\left\{\left|1-R_{xi}\right|,\left|N-R_{xi}\right|\right\}}\right)$$

where *WS* is a value of similarity coefficient, *N* is a length of ranking and *Rxi* and *Ryi* indicate the place in the ranking for *ith* element in, respectively, ranking *x* and ranking *y*.

For the second level (Table 8), the ranking is same for both applied methods; however, there is a difference in the third level (Table 9), where *WS* = 0.8869. This means that the similarity is significantly different which explains the superiority of the proposed approach.

## 4. Discussion

Frequent lane changes by road users can cause serious traffic issues. It has been considered very important to identify and prioritize the critical factors which could affect the frequency of lane changes. For evaluation purposes, the study utilized the combined AHP–BWM model to analyze the specified factors in a three-level hierarchical arrangement. This proposed model performs a better evaluation with a lower number of PCs than the conventional AHP. The final weights obtained from the conducted model are highly consistent as the adopted model gives more reliable results than the conventional AHP approach. In the conventional AHP, the consistency ratio for clusters consists of five elements or more as a measure to examine if the comparisons are consistent or not. While, in the BWM, the consistency ratio is used to check the degree of reliability. The current study adopted the input-based consistency threshold to measure the consistency of comparisons. Furthermore, the adopted model facilitates specialists in understanding the entire evaluation procedure. This consideration was justified by our survey results. The proposed combined AHP–BWM approach could help decision-makers to focus on highlighted significant factors affecting frequent lane-changing to improve road safety. Previous studies recommended the application of lane-change assisting systems to improve active safety and reduce traffic accidents due to frequent lane-changing [72].

Achieving sustainable traffic safety in the real world is a complicated issue that includes a large number of criteria and the interdependent characteristics of which can vary for all significant traffic engineering elements (e.g., the human, road and vehicles). Another fact to reflect upon is that decision-making concerning traffic safety should be approached by decision-makers via different routes. Hence, while the optimum solution to a problem is very challenging to find out, finding a solution for target levels or objectives is a better decision. These facts make it difficult to resolve the problem of attaining a sustainable traffic safety using traditional MCDM approaches. Thus, decision-making should consider the complexity of the real world. Some authors [73,74,75,76] utilized a new hybrid MCDM system (DEMATEL-based ANP with VIKOR) in an effort to resolve the limits of traditional MCDM approaches and attain sustainable development tactics in other fields. In the study [77], a new hybrid model which integrates the fuzzy step-wise weight assessment ratio analysis (FSWARA) and the fuzzy best–worst method (FBWM) was created for the selection of equipment in a container terminal. Furthermore, the authors in [78] utilized a fuzzy pivot pairwise relative criteria importance assessment (Fuzzy PIPRECIA) technique to evaluate and rank the road transportation risk elements in the Giresun province.

## 5. Conclusions

The assessment of transportation and traffic risk is a very common and important procedure that can aid participants in avoiding conflicts and risk conditions. The findings of this research highlighted the most significant factors which could cause frequent lane-change behavior and further contribute to a negative impact on road safety. Therefore, this categorization of factors will help stakeholders to decide the strategic policy for safe lane-changing to improve road safety. Considering the high-level significance of traffic volume and average speed for lane-changing, the study results depict that there is a need for the advancement of more efficient countermeasures to be adopted by traffic administration departments. Therefore, it is essential to promote safety policies, which can reduce the high traffic volume and speed variation. In addition, the study recommends the use of developed vehicle technologies such as vehicle-to-vehicle communication and automatic vehicles (AVs) to concentrate on the collaborative contact between vehicles which may mitigate these frequent lane-changing issues. This study considers the factors affecting lane-changing based on their significance found in previous studies. However, advanced simulation tools can be utilized to consider the important factors affecting frequent lane-changing. Furthermore, an advanced comprehensive study needs to be performed on a large extent by conducting a sensitivity analysis to check the constancy of the ranking achieved by the recommended methods. Moreover, the use of the fuzzy AHP approach should be considered to incorporate uncertainty and ambiguity into the evaluation procedure based on subjective decisions. Therefore, the individual can focus on the presentation of several types of fuzzy set theory for solving the issue. In addition, future research can be performed to assess the relationship between socio-demographic characteristics and their involvement in frequent lane-changing.

## Figures and Tables

**Table 1:** Sample characteristics of participants.

Variables	Frequency	Percentage (%)
*N*	49	100
***Age (years)***		
18–30	17	35
31–50	22	45
51 above	10	20
***Gender***		
Male	38	78
Female	11	22
***Duration of driving license (years)***		
01–05	13	27
06–15	28	57
16–30	8	16
***Education level***		
Bachelor’s degree	20	41
MSc/PhD	29	59
***Working status***		
Student	22	45
Job	27	55

**Table 2:** DBQ survey example for level 1 (AHP model).

Compare the Importance of Specified Factors with Respect to Frequent Lane-Changing																		
Traffic Characteristics	9	8	7	6	5	4	3	2	1	2	3	4	5	6	7	8	9	Human
Traffic Characteristics	9	8	7	6	5	4	3	2	1	2	3	4	5	6	7	8	9	Road Characteristics
Traffic Characteristics	9	8	7	6	5	4	3	2	1	2	3	4	5	6	7	8	9	Light conditions
Human	9	8	7	6	5	4	3	2	1	2	3	4	5	6	7	8	9	Road Characteristics
Human	9	8	7	6	5	4	3	2	1	2	3	4	5	6	7	8	9	Light conditions
Road Characteristics	9	8	7	6	5	4	3	2	1	2	3	4	5	6	7	8	9	Light conditions

**Table 3:** DBQ survey (BWM model).

Please, Select the Most Important Factor from Specified “Traffic Characteristics” Factors and Compare It with Other Factors by Considering Lane-Changing Phenomenon Using a (1–9) Judgment Scale					
Traffic Characteristics	Traffic Volume	Traffic Composition	Following Distance	Speed	Vehicle Type
Most important factor: (………….…)					
Less important factor: (………….…)					

**Table 4:** Saaty’s 1–9 scale for evaluating factors in the PCM.

Numerical Judgment Scale	Verbal Judgment Scale (Definition)
1	Equal significance
2	Weak importance
3	Moderate importance
4	More than moderate importance
5	Strong significance
6	More than strong significance
7	Very strong importance
8	Extremely important
9	Absolute significance

**Table 5:** Random index based on matrix dimensions (Saaty, 1977).

*n*	3	4	5	6	7	8	9	10
RI	0.58	0.9	1.12	1.24	1.32	1.41	1.45	1.49

**Table 6:** The threshold for different combinations of scaling and number of factors employing an input-based consistency measurement.

Scale	Factors						
	3	4	5	6	7	8	9
3	0.1667	0.1667	0.1667	0.1667	0.1667	0.1667	0.1667
4	0.1121	0.1529	0.1898	0.2206	0.2577	0.2577	0.2683
5	0.1354	0.1994	0.2306	0.2546	0.2716	0.2844	0.2960
6	0.1330	0.1990	0.2643	0.3044	0.3144	0.3221	0.3262
7	0.1294	0.2457	0.2819	0.3029	0.3144	0.3251	0.3403
8	0.1309	0.2521	0.3154	0.3108	0.3408	0.3620	0.3657
9	0.1359	0.2681	0.3337	0.3517	0.3517	0.3620	0.3662

**Table 7:** Factors weight scores based on the AHP–BWM model.

Level 1		Level 2		Level 3	
Factor	Weight	Factor	Weight	Factor	Weight
Main factors		Traffic characteristics		Speed	
Traffic characteristics	0.5148	Traffic volume	0.3440	Average speed	0.5148
Human	0.2134	Traffic composition	0.1075	Speed limit	0.4852
Road characteristics	0.1834	Following distance	0.1381	Vehicle type	
Light conditions	0.0884	Speed	0.2954	Light vehicle	0.7068
		Vehicle type	0.1150	Heavy vehicle	0.2932
		Human		Road type	
		Carelessness	0.2475	Urban	0.3026
		Illiteracy	0.1099	Rural	0.6974
		Violation of rules	0.3859	Road surface	
		Training	0.2566	Dry	0.7199
		Road characteristics		Wet	0.2801
		Road type	0.5969		
		Road surface	0.2203		
		Grade	0.1828		
		Light conditions			
		Daytime light	0.8396		
		Night light	0.1604		

**Table 8:** Comparisons of ranks for the factors in the second level based on the AHP–BWM model and conventional AHP.

Factor	Final Weight of AHP–BWM	Rank	Final Weight of AHP Approach	Rank
Traffic volume	0.1771	1	0.1819	1
Traffic composition	0.0554	8	0.0578	8
Following distance	0.0711	6	0.0690	6
Speed	0.1521	2	0.1425	2
Vehicle type	0.0592	7	0.0635	7
Carelessness	0.0528	10	0.0528	10
Illiteracy	0.0235	13	0.0235	13
Violation of rules	0.0824	4	0.0824	4
Training	0.0548	9	0.0548	9
Road type	0.1095	3	0.1095	3
Road surface	0.0404	11	0.0404	11
Grade	0.0335	12	0.0335	12
Daytime light	0.0742	5	0.0742	5
Night light	0.0142	14	0.0142	14

**Table 9:** Comparisons of ranks for the factors in the third level based on the AHP–BWM model and conventional AHP.

Factor	Final Weight of AHP–BWM	Rank	Final Weight of AHP Approach	Rank
Average speed	0.0783	1	0.0734	2
Speed limit	0.0738	3	0.0691	3
Light vehicle	0.0418	4	0.0449	4
Heavy vehicle	0.0174	7	0.0186	7
Urban	0.0331	5	0.0331	5
Rural	0.0764	2	0.0764	1
Dry	0.0291	6	0.0291	6
Wet	0.0113	8	0.0113	8

## Data Availability

All the essential data are presented in the manuscript.
